# Single-cell RNA sequencing reveals distinct chondrocyte states in femoral cartilage under weight-bearing load in Rheumatoid arthritis

**DOI:** 10.3389/fimmu.2023.1247355

**Published:** 2023-08-16

**Authors:** Mingyue Yan, Zewen Sun, Junjie Wang, Haibo Zhao, Tengbo Yu, Yingze Zhang, Tianrui Wang

**Affiliations:** ^1^ Department of Orthopedics, The Affiliated Hospital of Qingdao University, Qingdao, Shandong, China; ^2^ Institute of Sports Medicine and Health, Qingdao University, Qingdao, Shandong, China; ^3^ Department of Orthopedic Surgery, Qingdao Hospital, University of Health and Rehabilitation Sciences (Qingdao Municipal Hospital), Qingdao, Shandong, China; ^4^ Department of Orthopedics, The Third Hospital of Hebei Medical University, Shijiazhuang, Hebei, China

**Keywords:** Rheumatoid arthritis, chondrocyte, single-cell RNA sequencing, weight-bearing region, immune

## Abstract

**Introduction:**

Rheumatoid arthritis (RA) is a common autoimmune joint disease, the pathogenesis of which is still unclear. Cartilage damage is one of the main manifestations of the disease. Chondrocytes are the main functional component of articular cartilage, which is relevant to disease progression. Mechanical loading affects the structure and function of articular cartilage and chondrocytes, but the effect of weight bearing on chondrocytes in rheumatoid arthritis is still unclear.

**Methods:**

In this paper, single-cell RNA sequencing (scRNA-seq) was performed on collected cartilage from the weight-bearing region (Fb group) and non-weight-bearing region (Fnb group) of the femur, and the differences between the Fb and Fnb groups were analyzed by cell type annotation, pseudotime analysis, enrichment analysis, cell interactions, single-cell regulatory network inference and clustering (SCENIC) for each cell type.

**Results:**

A total of 87,542 cells were analyzed and divided into 9 clusters. Six chondrocyte subpopulations were finally identified by cellular annotation, and two new chondrocyte subtypes were annotated as immune-associated chondrocytes. The presence of each chondrocyte subpopulation and its distribution were verified using immunohistochemical staining (IHC). In this study, the atlas of femoral cartilage in knee rheumatoid arthritis and 2 new immune-related chondrocytes were validated using scRNA-seq and IHC, and chondrocytes in the weight-bearing and non-weight-bearing regions of the femur were compared. There might be a process of macrophage polarization transition in MCs in response to mechanical loading, as in macrophages.

**Conclusion:**

Two new immune-associated chondrocytes were identified. MCs have contrasting functions in different regions, which might provide insight into the role of immune and mechanical loading on chondrocytes in the development of knee rheumatoid osteoarthritis.

## 1 Background

Rheumatoid arthritis (RA) is a common chronic progressive autoimmune joint disease, the onset and progression of which are closely related to immune cells, synoviocytes, and osteoclasts (1), and the pathogenesis has not been fully investigated. Knee rheumatoid arthritis is characterized by knee pain and limited mobility, with the main pathological features including synovitis, progressive bone erosion, and cartilage damage (2). It has been shown that chondrocyte proliferation, apoptosis, and autophagy are associated with the progression of RA disease (3), suggesting that a deeper understanding of cartilage would facilitate a better exploration of the pathogenesis of RA.

Articular cartilage is an important connective tissue on the joint surface, with a smooth and elastic surface that reduces joint friction and motion shock. Cartilage tissue is mainly composed of chondrocytes and secreted extracellular matrix, but abnormal mechanical loading can affect the metabolic balance of chondrocytes and have an impact on the catabolism of the cartilage extracellular matrix (4). It has also been demonstrated that mechanical overload not only affects chondrocyte proliferation but also induces chondrocyte apoptosis (5, 6). At the same time, stress stimulation from joint motion is one of the important external factors regulating cartilage growth and development, and lack of stress stimulation increases chondrocyte IL-4 or IL-10 levels, which in turn exacerbates cartilage breakdown (7). In this study, cartilage damage was found to be more severe in the weight-bearing region of the femur during knee replacement surgery in RA patients, and it was hypothesized that assessment of chondrocytes in both weight-bearing and non-weight-bearing regions might provide further insight into the function of chondrocytes and facilitate an in-depth exploration of the pathogenesis of RA.

Single-cell RNA sequencing (scRNA-seq) enables the analysis of cells in tissues at single-cell resolution, filling the gap of high-throughput transcriptome technologies that cannot be sequenced precisely to cell type. This technique has been widely used in autoimmune diseases to reveal cellular heterogeneity and identify pathogenic cell subpopulations in a wide range of immune inflammation-related tissues (8). A study found that the proportion of helper T cells and activated T cells in the synovium of rheumatoid arthritis is higher than that of osteoarthritis(OA) (9). Up to now, single-cell studies in RA have mostly focused on tissues such as peripheral blood and synovium (10, 11), and relatively few studies have been conducted on chondrocytes. Tang et al. performed the first single-cell sequencing of chondrocytes in OA and identified subpopulations of chondrocytes such as fibrocartilage chondrocyte (FC), effector chondrocyte (EC), homeostatic chondrocyte (HomC), regulatory chondrocyte (RegC) and cartilage progenitor cells (CPC) (12). Our team’s previous single-cell study of healthy cartilage in the ankle joint also identified two new subpopulations of chondrocytes, which were named metal ion related chondrocytes (MirCs) and splicing chondrocytes (SpCs) (13).

Mehmet et al. proposed that analysis of differential genes between damaged and undamaged cartilage could help determine the exact cause of focal cartilage damage (14). Therefore, in this study, cartilage from the weight-bearing and non-weight-bearing regions of the femur of RA patients was taken separately and sequenced with scRNA-seq to explore the function of chondrocyte subpopulations as well as to complement and validate the previously identified chondrocyte subtypes. This study hopefully provides insight into the effects of mechanical loading and immunological factors on chondrocytes and offers a reference for further exploration of the pathogenesis and genetic markers of RA.

## 2 Methods

### 2.1 Volunteers screening and samples selection

**Figure 1:**
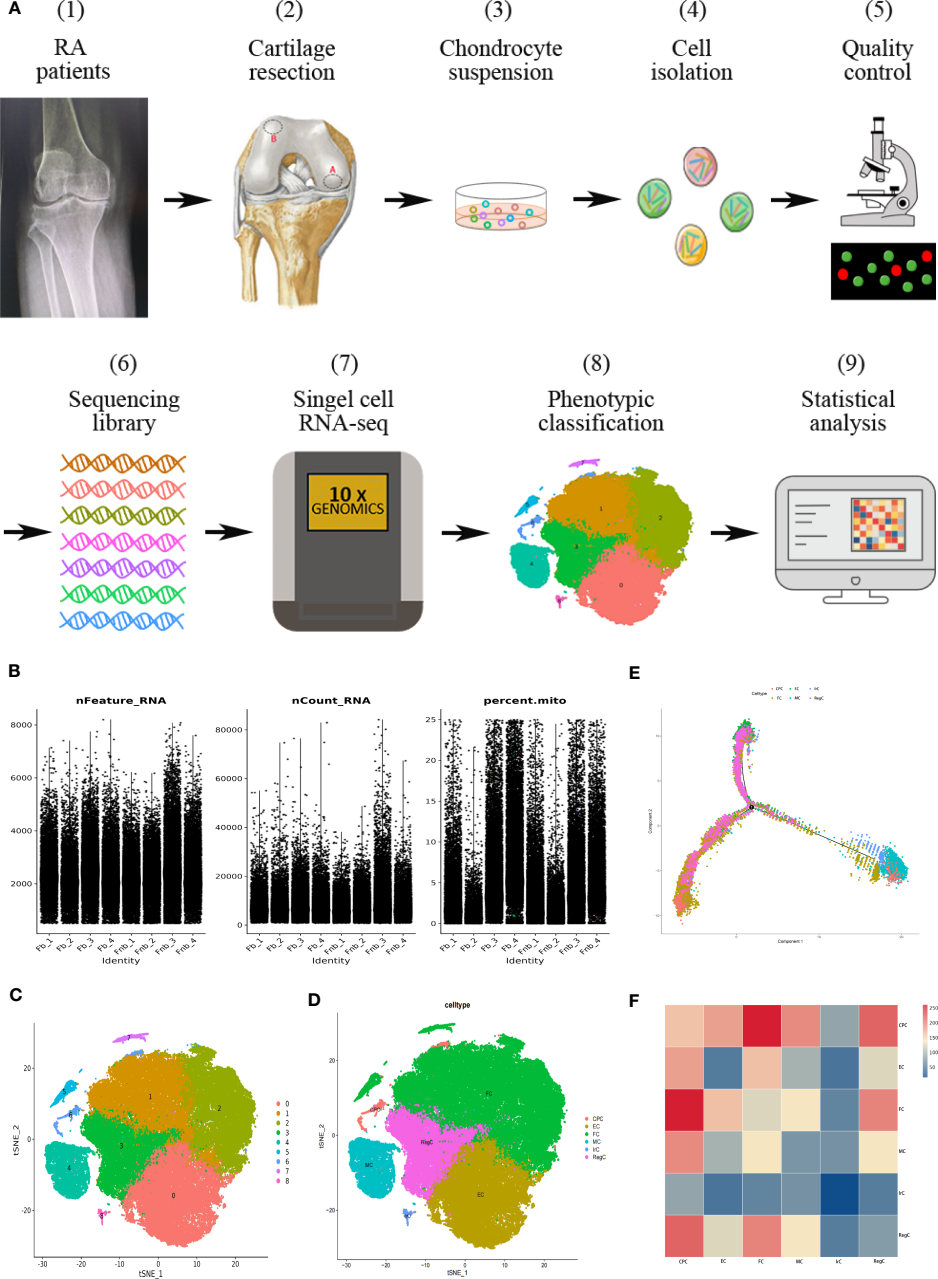
Single-cell RNA-seq of human RA cartilage chondrocytes. **(A)** Schematic workflow of the experimental strategy. The circles represented the location of the cartilage samples taken, with region A being the weight-bearing region and region B being the non-weight-bearing region. **(B)** Quality control conditions. **(C)** Clustering results. **(D)** Cell type annotation results. **(E)** Pseudotime analysis of each cell type using the Moncle function. Trajectory analysis was performed for the entire development line, and the color of each point represented the order of cell growth and differentiation. **(F)** The interaction strength of intercellular communication between cell types. The color of the rectangle represented the probability of the role. CPC, cartilage progenitor cells; EC, effector chondrocyte; FC, fibrocartilage chondrocyte; IrC, inflammatory related chondrocyte; MC, macrophage chondrocyte; RegC, regulatory chondrocyte; RA, rheumatoid arthritis; t-SNE, t-distributed stochastic neighbor embedding.

The flow chart of the study is shown in **Figure 1A**. In this study, the volunteers were taken from patients who had no history of knee trauma and were diagnosed with RA, with proposed knee replacement in our hospital. We took cartilage from the weight-bearing and non-weight-bearing regions of the joint on the discarded femur from the knee replacement surgery. The locations of the obtained cartilage samples are shown as red circles in **Figure 1A** (2), with region A being the weight-bearing region and region B being the non-weight-bearing region. We cumulatively obtained a total of 7 groups of surface cartilage from the weight-bearing region of the femur (called the Fb group) and the non-weight-bearing region (called the Fnb group) from 7 patients, and performed scRNA-seq on 4 groups (8 samples) and immunohistochemical staining (IHC) analysis on 3 groups (6 samples). The study was approved by the Institutional Ethics Review Committee of the Affiliated Hospital of Qingdao University(QYFY WZLL 27403), and all donor patients signed a written informed consent.

### 2.2 Cartilage zoning, segmentation, and single-cell suspension preparation

All cartilage tissue was isolated within 3 hours of the knee replacement osteotomy, and two experienced clinicians evaluated the cartilage and positioned the weight-bearing and non-weight-bearing regions. The surgically discarded bone tissue was then placed in sterile saline and transferred to an ultra-clean table to segment the cartilage tissue, ensuring that the excised cartilage tissue did not retain any subchondral bone. The cartilage tissue was then transferred to sterile Petri dishes placed on ice in phosphate-buffered saline (PBS). Each tissue sample was approximately 0.25 × 1 × 2 cm in size and weighed approximately 0.5 ~ 0.6 g. The cartilage samples were cut into 0.5 mm^3^ and rinsed twice with PBS. The digestion solution (0.2% collagenase II and 0.25% EDTA-trypsin) was preheated in a water bath at 37°C, into which the tissue blocks were shaken at 100 rpm for 20 min at 37°C to obtain a single cell suspension. Afterward, PBS containing 10% fetal bovine serum was added to stop the digestion. The obtained cell suspension was filtered through a 70 μm cell strainer and centrifuged at 300 × g for 5 min at 4°C. Dead cells were removed using the Miltenyi® kit (MACS 130-090-101). Finally, the cell suspension was resuspended by centrifugation at 300 × g for 3 min at 4°C 2 times. The overall cell viability was confirmed over 85% using Tissue Blue and the cells in the single-cell suspension were counted by an automated cell counter at a density of 700 - 1200 cells/μl.

### 2.3 10× Genomics scRNA-seq library preparation and cell clustering

The prepared single-cell suspensions were added to 10x chromium, and the single cells were extracted and amplified according to the instructions of the 10X Genomics Chromium Single-Cell 3 kit (V3). The cDNA library was constructed following standard procedures. The library was sequenced on an Illumina NovaSeq 6000 sequencing system with a minimum of 20,000 reads per cell by LC-Bio Technology Co.Ltd (Hangzhou, China).

Sequencing files were converted to a FASTQ format by Illumina bcl2fastq software (version 2.20), and the sample data were processed and counted using the Cell Ranger pipeline (version 4.0.3). The scRNA-seq data were decoded using the Ensembl GRCh38/GRCm38 reference genome, and the output data were then loaded into the SeuratR package (version 3.1.1) for normalization, reduction, and clustering. Genes expressed in less than 3 cells were not incorporated into the analysis. In this program, the number of genes expressed in a single cell should be more than 500, and the proportion of genes of mitochondrial DNA origin should be less than 25%. Since the presence of ribosomal genes affects the results of cell clustering, we used the Rsubread, edgeR, and scater packages to remove the sequences of ribosomal genes. The samples were integrated using the RunHarmony in Seurat. Perform dimensionality reduction and visualization analysis of the data using the RunTSNE in Seurat. The FindMarkers in Seurat was used to identify marker genes expressed in more than 10% of cells in each cluster and to screen for differentially expressed genes (DEGs) between the Fb and Fnb groups. Use the featureplot to visualize the expression levels of marker genes in the t-SNE plot. The ggplot2 R package was used to visualize the results of the DEG analysis.

### 2.4 Gene ontology and Kyoto encyclopedia of genes and genomes pathway enrichment analyses

GO enrichment analysis is a commonly used bioinformatics tool to determine whether a set of genes is enriched in a biological process or function, which is used to annotate genes and analyze their biological processes. The KEGG analysis based on the KEGG database is commonly used for functional annotation and enrichment analysis of genes and metabolic pathways, which helps to understand the interactions between biomolecules and metabolic pathways. We performed GO and KEGG enrichment analysis using Metascape (http://metascape.org/gp/#/main/step1) to gain insight into the biological function of each cluster, with a P value < 0.05 indicating significant results.

### 2.5 Identification of different cell types

DEGs with high log2FC values and high specificity were selected as marker genes for each cluster. The marker genes were normalized for each cell type using the pheatmap package, after which they were clustered and heatmaps were drawn. The analyzed marker genes were compared with the list of already validated marker genes to identify the cell types (12, 13). The results of GO and KEGG pathway enrichment analysis were also taken into account when annotating cell types. The results of the enrichment analysis were visualized using the ggplot2 package.

### 2.6 Protein-protein interaction network construction and hub gene identification

Search Tool for the Retrieval of Interacting Genes (STRING; http://string-db.org)(version 11.5) is an online tool for analyzing gene coding protein interactions. Cytoscape (version 3.9.1) is an open-source network analysis software that can be used for biological network analysis and visualization of the results. We used STRING to construct a PPI network of DEGs between the Fb and Fnb groups. The information obtained from STRING was imported into Cytoscape, and the top 10 hub genes were identified by cytoHubba.

### 2.7 Pseudotime analysis

Seurat objects with annotations were converted to readable cds objects. Cells were aligned along the simulated cell developmental trajectory, and the orderCells function was used to show cells’ presumed position in the Monocle. Different colors represented different cell subpopulations.

### 2.8 Cell-cell interactions

We use the CellChat R package to analyze cellular interaction networks and to calculate probability values for cell-cell interactions by combining single-cell expression profiles with known ligands and receptors. Seurat objects with annotations need to be converted to CellChat objects. Over-expressed ligands or receptors in one cell type were identified using the IdentifyOverExpressedGenes function. Then, gene-expression data are projected into the protein interaction network using the projectData function in CellChat. Use the function of IdentifyExpressedInteractionCellChat to screen for ligand-receptor interactions when either the ligand or its receptor was overexpressed.

### 2.9 Single-cell regulatory network inference and clustering

SCENIC is a single-cell RNA sequencing analysis tool for resolving transcriptome data from individual cells to characterize transcription factor activity in individual cells. Seurat objects with annotations were imported to run Scenic (version 0.9.1). Use GENIE3 to extrapolate gene regulatory networks from gene expression data. The TF targeting relationship with the gene was verified with the help of RcisTarget software. Then, cells with active gene regulatory networks were identified using AUCell software, and regulons were scored for their Area Under Curve (AUC) by binarizing specific regulons. The RSS score of the TF was obtained by the calcRSS and ranked by value to determine the Top TF for each cell type. The differentially expressed transcription factors were visualized using the pheatmap function.

### 2.10 Statistical analysis

The Wilcoxon rank sum test and independent samples t-test were selected for data analysis of the number of different cell subpopulations between groups according to the characteristics of the data format. R packages stats and car were used to compare the differences in cell numbers between the Fb and Fnb groups, and the data were visualized with the ggplot2 package. P values < 0.05 were considered statistically significant.

### 2.11 IHC

Immunohistochemical staining(IHC) was performed as follows. Distal femoral cartilage tissue was trimmed and then incubated in 4% buffered paraformaldehyde fixative for 48h. The tissues were then incubated in EDTA for 3 months for decalcification, and the decalcification effect was detected using a needle. Afterward, the tissues were embedded in paraffin, and the paraffin samples were dewaxed and rehydrated with xylene and ethanol. Subsequently, samples were incubated in 3% hydrogen peroxide for 10 minutes to block endogenous peroxidase activity. Antigen repair was performed by treatment with 3% bovine serum albumin at room temperature for 30 min and digestion with pepsin. Later, the samples were incubated with primary antibodies (specific names of these primary antibodies are mentioned in the results section) for 12 h at 4°C, followed by secondary antibody administration for 50 min at room temperature. Finally, the samples were counterstained with DAB and hematoxylin nuclei for about 3 min. The stained sections were visualized and imaged with a vertical microscope (Nikon Eclipse Ci) and an imaging system (Nikon Digital Sight DS-FI2) with white light. IHC staining of cartilage sections was scored blindly by experienced pathologists using a light microscope. The widely accepted German semi-quantitative scoring system was used, taking into account the range of staining strength and staining area. Staining intensity: 0, no coloring; 1, light yellow; 2, brown-yellow; 3, brown-brown. Stained area: 0, <5%; 1, 5%-25%; 2, 25%-50%; 3, 51%-75%; 4, >75%. These two scores were multiplied together as the final score. Comparative analysis of IHC staining scores for each layer of cartilage tissue was performed using the Kruskal-Wallis test for within-group differences and the Wilcoxon rank sum test for between-group differences. R package ggplot2 was used for data visualization.

## 3 Results

### 3.1 Single-cell profiling of human RA cartilage chondrocytes

Ultimately, 87,542 cells were analyzed and divided into cluster0-8. The basic quality control conditions were as shown in **Figure 1B**. **Figure 1C** displayed the clustering results and summary results of the cell type annotation. 9 cell clusters were combined and annotated into 6 chondrocyte subpopulations, including CPC, EC, FC, IrC(inflammatory related chondrocyte), MC(macrophage chondrocyte), and RegC (**Figure 1D**). The results of the pseudotime analysis were shown in **Figure 1E**. EC was selected as the initial stage according to its distribution. The results of cellular interactions were shown in **Figure 1F**, indicating that there might be strong interactions between CPC and FC, and RegC. The results of cell interactions were provided in **Supplementary Data** (**Supplemental Data Table 1**).

### 3.2 Identification of human RA cartilage chondrocyte populations and TF regulation

**Figure 2:**
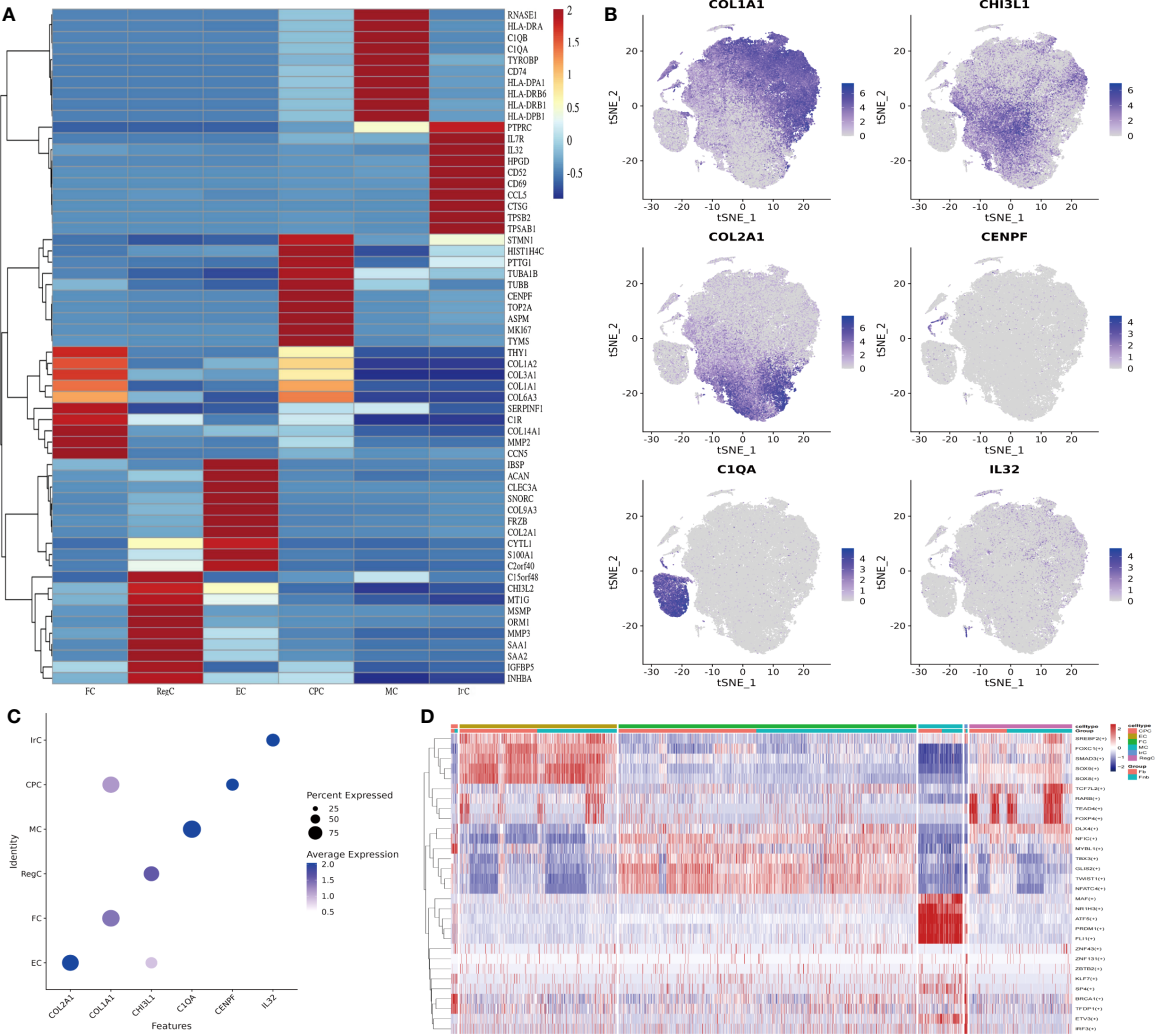
Identification of chondrocyte subpopulations. **(A)** Heatmap revealing the scaled expression of differentially expressed genes for each cell type. Specific representative genes in each chondrocyte subset were highlighted along the right margin. The color scheme is based on z-scores. **(B)** Clustering plots of Marker genes in cell subpopulations. **(C)** DotPlot of cell Marker genes. **(D)** Differences in transcription factor expression between different groups and cell types, with colors indicating transcription factor AUC values in different cells. Fb, femur weight-bearing region; Fnb, femur non-weight-bearing region. CPC, cartilage progenitor cells; EC, effector chondrocyte; FC, fibrocartilage chondrocyte; IrC, inflammatory related chondrocyte; MC, macrophage chondrocyte; RegC, regulatory chondrocyte; RA, rheumatoid arthritis; t-SNE, t-distributed stochastic neighbor embedding.

We collated the Top10 DEGs for different cell types (**Supplemental Data Table 2**) and plotted the gene expression heat map (**Figure 2A**). Gene expression clustering plots (**Figure 2B**) and DotPlot plots (**Figure 2C**) showed the specific distribution of marker genes. The cell interactions (**Figure 2D**) show the Top5 TF of each cell subpopulation, showing that KLF7 mainly affects CPC, SOX9 mainly affects EC, NFATC4 has some specificity in FC, MAF is more significant in MC, IRF3 is mainly associated with IrC and DLX4 has a greater effect on RegC.

**Figure 3:**
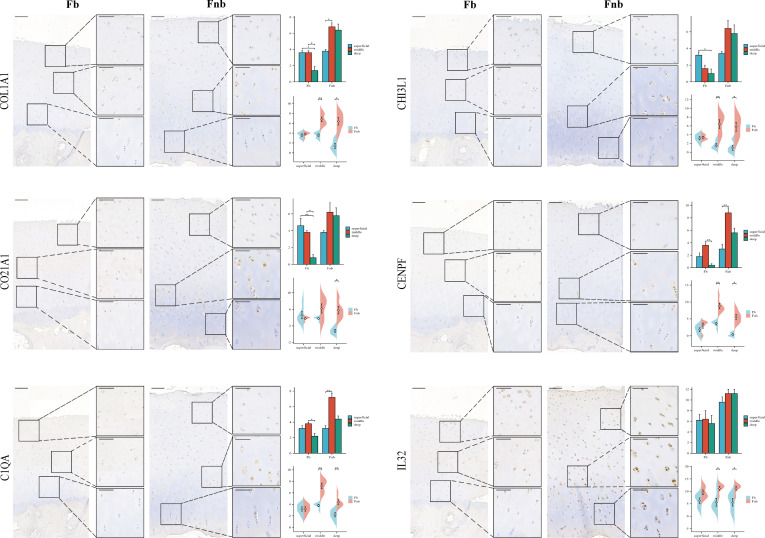
IHC results for specific markers for each cell type in different groups and layers. Scale bar, left, 200 µm; right, 100 µm. The scores of the indicated genes in the cartilage of both regions based on the immunohistochemistry assay are shown.*p<0.05, **p<0.01; otherwise, not significan Fb, femur weight-bearing region; Fnb, femur non-weight-bearing region.CPC, cartilage progenitor cells; EC, effector chondrocyte; FC, fibrocartilage chondrocyte; IrC, inflammatory related chondrocyte; MC, macrophage chondrocyte; RegC, regulatory chondrocyte.

IHC was performed to validate the gene markers in different chondrocyte types (**Figure 3**). FCs were distributed throughout the cartilage, slightly more in the middle layer and less in the weight-bearing region. RegCs were visible in all layers of cartilage, but there were relatively fewer in the weight-bearing region. ECs were similarly distributed throughout the cartilage, with fewer in the weight-bearing region, and the differences were mainly in the deep layer. CPCs were primarily found in the middle layer of cartilage, with significantly less in the middle and deep layers of the weight-bearing region. MCs were present throughout the cartilage, and there were higher concentrations in the middle layer. In the weight-bearing region, there were significantly fewer MCs in the middle and deep layers compared to the non-weight-bearing region. IrCs were more abundant in all layers of cartilage, similar to the distribution of MCs, with significantly lower numbers in the middle and deep layers of the weight-bearing region compared to the non-weight-bearing region.

**Figure 4:**
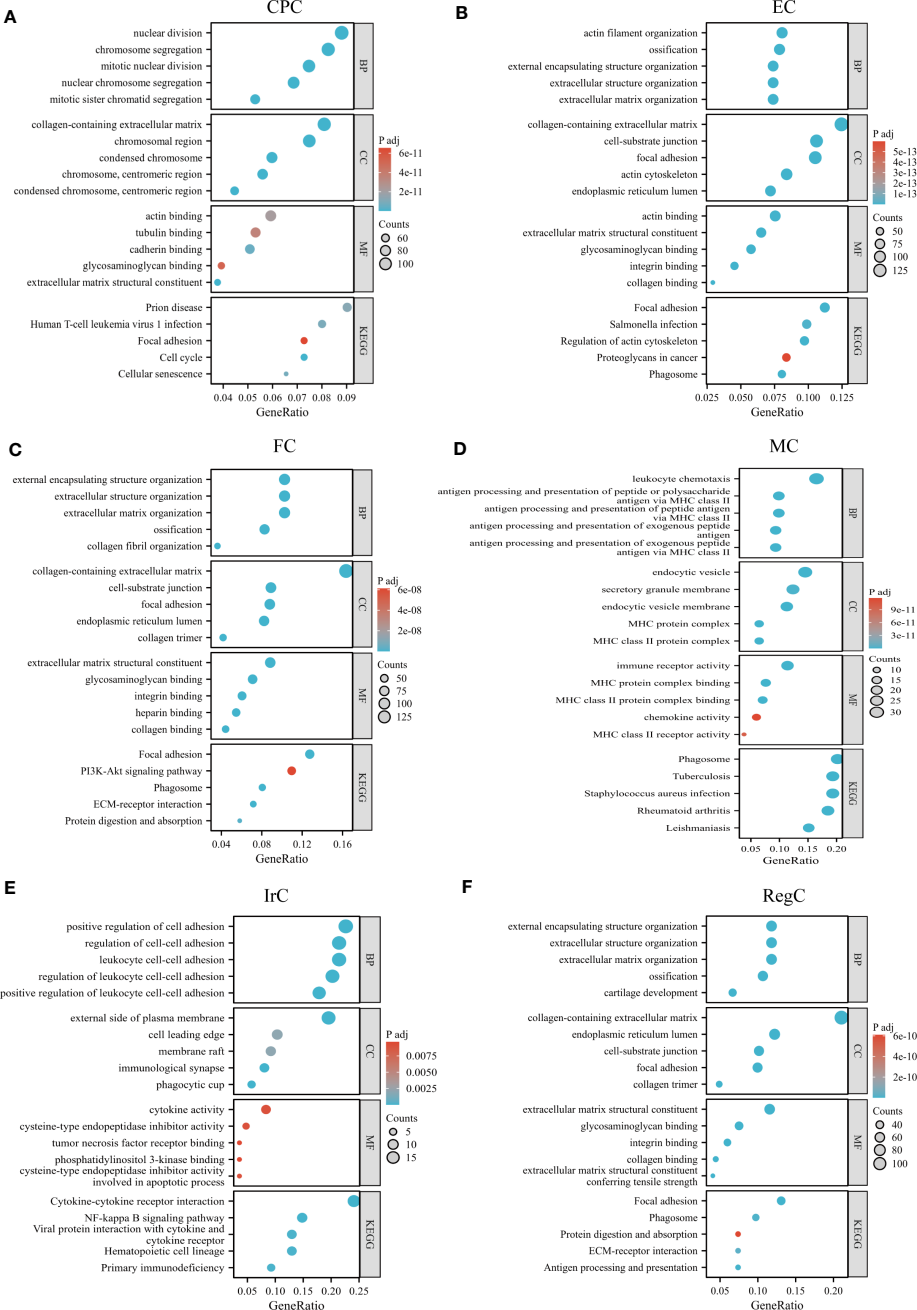
GO/KEGG enrichment analysis results of each cell type. **(A–F)** GO/KEGG enrichment results for different cell subpopulations, circle color indicates the p-value of each term, and circle size indicates the number of genes enriched in each term. CPC, cartilage progenitor cells; EC, effector chondrocyte; FC, fibrocartilage chondrocyte; IrC, inflammatory related chondrocyte; MC, macrophage chondrocyte; RegC, regulatory chondrocyte.

We performed GO/KEGG pathway enrichment analysis for each cell subpopulation of DEGs, and the detailed results were shown in **Supplemental Data Tables 3, 4**. The bubble plots showed the results of GO and KEGG enrichment analysis (**Figures 4A–F**). CPC was mainly associated with nuclear division, chromosomes, extracellular matrix structural constituents, and cell cycle. EC-related genes were mostly enriched in the collagen-containing extracellular matrix, ossification, and focal adhesion. FC was mainly enriched in extracellular structure organization and ECM-receptor interaction. RegC was mainly associated with cartilage development, collagen-containing extracellular matrix. Cluster4 was mainly associated with antigen processing and presentation, MHC class II protein complex, immune receptor activity, and KEGG suggesting a close relationship with phagosome and Rheumatoid arthritis. Cluster8 was enriched to the regulation of leukocyte cell-cell adhesion, immunological synapse, cytokine activity, and cytokine-cytokine receptor interaction. Our team combined the GO/KEGG enrichment results and marker gene annotated by Wang et al. on immune cells (15). The macrophage subpopulation(APOC1) was more matched with Cluster4 (**Supplemental Data Figure 1**). The function of cluster 8 was not clearly indicated, and we tentatively found that it was related to inflammatory processes such as leukocyte adhesion and cytokine receptors. Therefore, Cluster4 was named macrophage chondrocyte(MC), and cluster8 was named inflammatory related chondrocyte(IrC).

**Figure 5:**
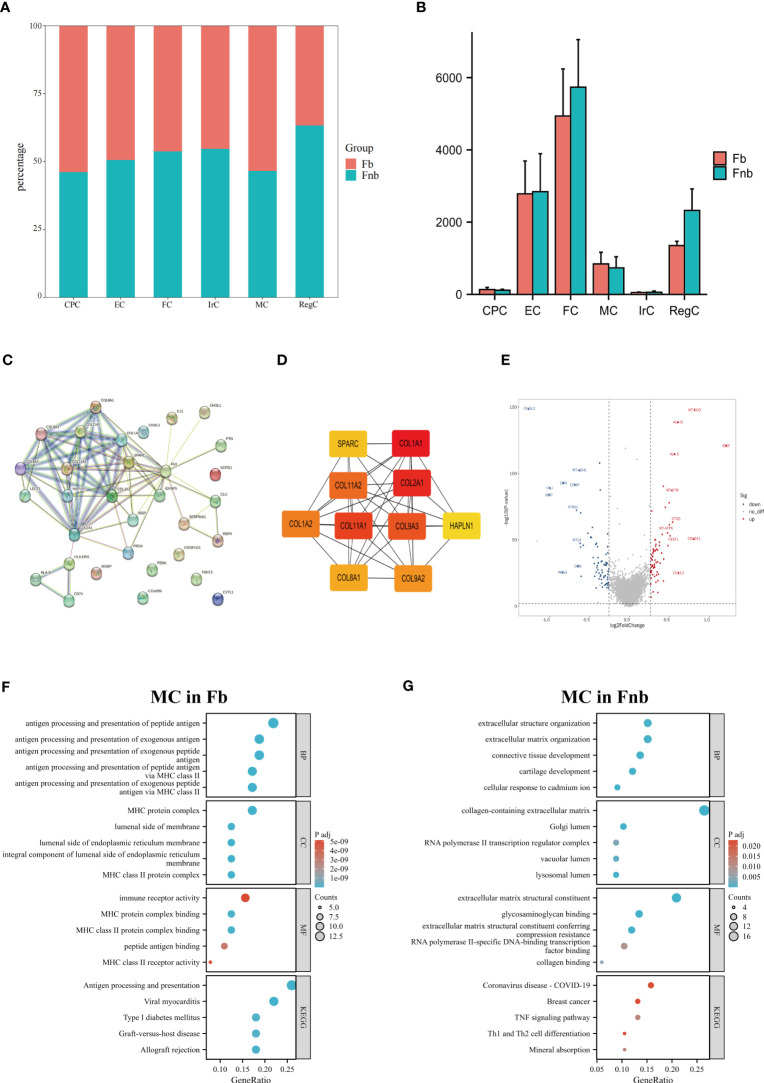
Results of differences analysis between Fb and Fnb. **(A)** Differences in the proportion of cell subpopulations. **(B)** Differences in the number of cell subpopulations. **(C)** The PPI network constructed by the String tool. Nodes represented proteins produced by protein-coding genes, and connections between nodes represented predicted protein interactions. **(D)** The top 10 Hub genes predicted by Cytoscape. **(E)** Volcano plot of MC DEGs between Fb and Fnb groups. The horizontal coordinate in the plot represented logFC and the vertical coordinate represented p-values. **(F–G)** Results of GO and KEGG enrichment analysis of MC between Fb and Fnb groups. The size of the circle indicated the number of enriched DEGs and the color represented the p-value. Fb, femur weight-bearing region; Fnb, femur non-weight-bearing region; GO, gene ontology; CPC, cartilage progenitor cells; EC, effector chondrocyte; FC, fibrocartilage chondrocyte; IrC, inflammatory related chondrocyte; MC, macrophage chondrocyte; RegC, regulatory chondrocyte.

We also calculated and compared the proportion of each cell type and cluster in the Fb and Fnb groups (**Figure 5A**; **Supplemental Data Figure 2**). We used the Wilcoxon rank sum test to compare the number of each cell subpopulation between the two groups (**Figure 5B**), and the characteristics of the data format were in **Supplemental Data Tables 5, 6**. The Fnb group had more RegCs, but this was not statistically significant. Based on the DEGs between the two groups (**Supplemental Data Table 7**). The volcano plot of DEGs between Fb and Fb was shown in **Supplemental Data Figure 3**. The DEGs were imported into String, an online tool for PPI network analysis (**Figure 5C**; **Supplemental Data Table 8**), after which the data were imported into Cytoscape, and the Top 10 Hub genes were identified using the cytoHubba plugin (**Figure 5D**). DEGs of MC between Fb and Fb were also analyzed (**Supplemental Data Table 9**). **Figure 5E** showed a volcano plot of DEGs in MC between Fb and Fb. GO and KEGG enrichment analyses were performed. (**Figures 5F, G**; **Supplemental Data Table 10**). The results showed that the MCs in the Fb group were mainly associated with antigen processing and presentation and MHC class II protein complex binding; while the MCs in the Fnb group were mainly enriched to cartilage development and extracellular matrix organization.

### 3.3 Identification of chondrocyte populations in MC and IrC

**Figure 6:**
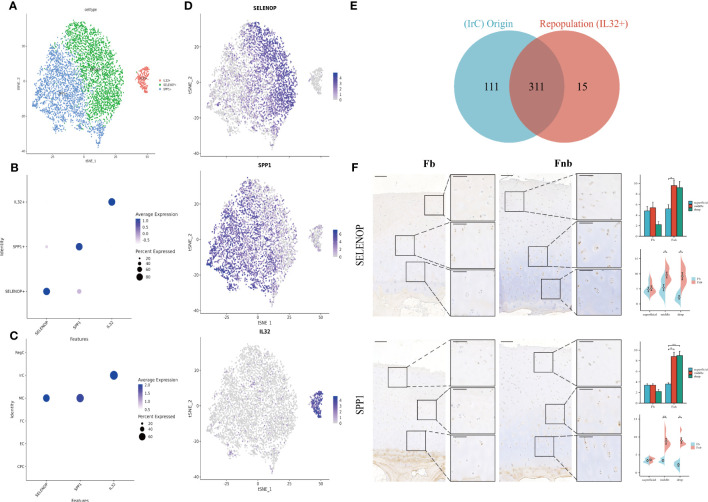
Marker genes for Immune-related chondrocytes. **(A)** Clustering plot of three cell subpopulations. **(B, C)** DotPlot of the cell marker genes. **(D)** A scatter plot of the cell marker genes, with the color of the dots representing expression levels. **(E)** Venn diagram for cell ID comparison of IrC and IL32+. The overlap represented the number of repetitive cells in the subpopulation. **(F)** IHC results for marker genes in different groups and layers. Scale bar, left, 200 µm; right, 100 µm. The scores of the indicated genes in the cartilage of both regions based on the immunohistochemistry assay are shown. *p<0.05, **p<0.01; otherwise, not significant. CPC, cartilage progenitor cells; EC, effector chondrocyte; FC, fibrocartilage chondrocyte; IrC, inflammatory-related chondrocyte; MC, macrophage chondrocyte; RegC, regulatory chondrocyte.

The two immune-related cell subpopulations, MC and IrC, were reclustered and 5981 cells were retained to obtain a total of 4 clusters. The expression clusters of marker genes and the results of enrichment analysis of cluster1(SPP1) and cluster3(AREG) in repopulation were similar(**Supplemental Data Figures 4, 5**; **Table 11**), so cluster1 and cluster3 were combined to obtain three subpopulations of chondrocytes, SELENOP+, SPP1+, and IL32+ (**Figure 6A**). The Dot Plot of the genes suggested that the marker genes were selected with specificity (**Figure 6B**), with SELENOP+ and SPP1+ positive cells mainly originating from MC. IL32+ cells were mainly derived from IrC (**Figure 6C**). Then we validated it by matching cell IDs. We found that 95% (311/326) of the cells in the IL32+ were from IrC (**Figure 6E**; **Supplemental Data Table 12** **)**. Expression clustering plots (**Figure 6D**) show the distribution of each cell type by a specific marker, validated using IHC (**Figure 6F**). The results suggest that SELENOP+(SELENOP) and SPP1+(SPP1) were distributed in the whole cartilage layer, mainly in the middle and deep layers, and less in the weight-bearing region.

**Figure 7:**
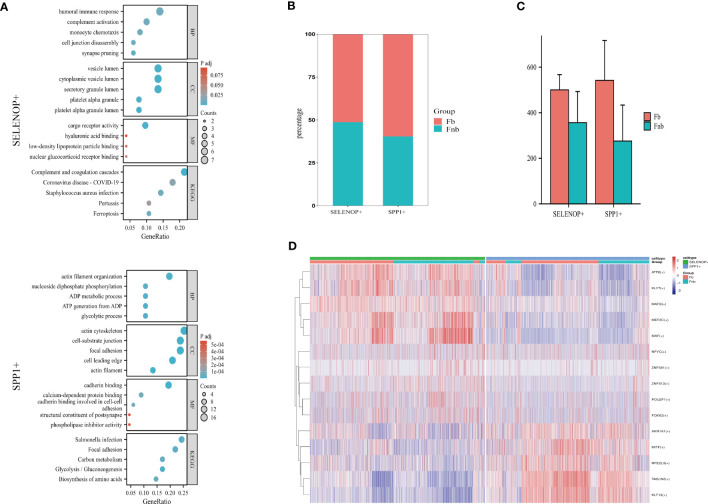
Characteristics of 3 clusters in Immune-related chondrocytes. **(A)** Results of GO and KEGG enrichment analysis for the three clusters. The size of the circles indicated the number of enriched DEGs, and colors represented p-values. **(B)** Differences in the proportion of cell subpopulations. **(C)** Differences in the number of cell subpopulations. **(D)** Differences in transcription factor expression between different groups and cell types. Colors indicate transcription factor AUC values in different cells. Fb, femur weight-bearing region; Fnb, femur non-weight-bearing region.

The GO and KEGG enrichment analysis of the two cell subpopulation marker genes (**Supplemental Data Table 13**) suggested that SELENOP+ MCs were associated with immune response, complement activation, and monocyte chemotaxis; SPP1+ MCs were associated with ATP generation and focal adhesion (**Figure 7A**). The proportion of each subpopulation of MC in the Fb and Fnb groups was calculated and compared(**Figure 7B**). We used the independent samples t-test to compare the number of each cell subpopulation between the two groups (**Figure 7C**), and the characteristics of the data format were in **Supplemental Data Tables 14, 15**. The proportion of SPP1+ cells in the Fb group was relatively high, but the statistical significance was not significant. **Figure 7D** showed the top5 TF of the 3 cell subpopulations, showing that MAF mainly affected SELENOP+ cells, while TAGLN2 had some specificity in SPP1+ cells.

## 4 Discussion

Rheumatoid arthritis (RA) is an autoimmune disease characterized by synovitis, bone erosion, and cartilage damage (progressive joint destruction), and its pathogenesis is not yet fully understood. Our team found heavy cartilage erosion in the weight-bearing region during RA knee replacement surgery. In this study, we investigated chondrocytes in the weight-bearing and non-weight-bearing regions of femoral cartilage in RA patients using single-cell sequencing technology and performed analysis and experimental validation of chondrocyte subsets and marker genes. It is worth mentioning that we identified immune-related subtypes of chondrocytes, new markers, and signaling pathways that may be involved in the pathogenesis of RA based on scRNA-seq analysis, hoping to provide some reference and help in the diagnosis and treatment of human RA.

Our sequencing analysis of femoral cartilage from RA patients screened for a total of six cell subsets, including two of our newly defined immune-related chondrocyte subsets (MC and IrC). The finding by Tang et al. that some of the RegC cells with high expression of CD74, CD86, and HLA-DPA1 might have immune cell functions during OA progression corroborates with the findings of MC and IrC in this study (12). The psuedotime analysis resulted in a binary branching structure (**Figure 1E**), where the EC is located at the beginning of cellular evolution on the graph, which is also consistent with the study by Tang et al. (12). CPCs are less frequent at the start of differentiation, accumulating mainly at the ends of branches and at the ends of trajectories. Notably, FC and RegC subpopulations are present throughout the developmental trajectory, with FC accumulating mainly at the end of branches and RegC significantly less at the end of the trajectory. In contrast, MC and IrC accumulate mainly at the end of the differentiation trajectory. We also performed an in-depth analysis of cellular interactions (**Supplemental Data Table 1**), with CPC interacting relatively closely with cellular subpopulations such as FC/RegC/MC (**Figure 1F**). CD74, a receptor-related molecule associated with the antigen presentation process, has a high degree of activation between MC and six cell types, including itself, where CD74-MIF may play a role in regulating macrophage migration. It has been suggested that sufficient ferritin can be loaded with large amounts of iron ions and can lead to further oxygen radical damage (16). Our study also found that the molecules SCARA5 and FTL/FTH1, which are associated with iron ion transport, have a greater impact on the interaction species between MC/IrC and various other cell subpopulations, and perhaps the iron ion and RA relationship deserves further exploration. Molecular pairs of osteogenesis-related genes such as FN1 and integrin (α4β1) activate communication between RegC/EC/CPC, suggesting that these cell subpopulations may have a role in chondrocyte differentiation and collagen synthesis (17).

During RA progression, autoantigens would activate a specific immune response, with increased secretion of inflammatory cytokines and infiltration of synovial joints, resulting in arthritic symptoms (18). It was shown that joint disease with cartilage damage was closely related to immunological elements and it was recognized that the immune response was a key factor influencing cartilage repair (19, 20). During cartilage repair, immune cells such as macrophages could secrete anti-inflammatory factors which in turn promoted cartilage repair (21). However, the persistence of pro-inflammatory factors could also lead to chondrocyte death and accelerate the degradation of the cartilage (22). Therefore, understanding the relationship between chondrocytes and the immune response at the single-cell level could help to unravel the mechanisms underlying RA disease progression.

In this study, we identified two new subpopulations of immune chondrocytes. The MC subpopulation, located in the whole cartilage layer and more in the middle layer, showed high immunological activity and was mainly involved in immunological processes such as antigen processing and presentation, MHC class II protein complex, and immune receptor activity. KEGG suggested a close relationship with Rheumatoid arthritis. Our sequencing results revealed that HLA family genes such as HLA-DRA and HLA-DRB1 are highly expressed in MC, and the enrichment results of these genes showed an association with RA disease (**Supplemental Data Tables 3, 4**). Human leukocyte antigens (HLA) have gained some attention as antigen-presenting receptors, and many researchers suggested that HLA-DRB1 might be strongly associated with RA and influence the severity of the disease (23, 24). The interaction between macrophages and chondrocytes was also found in OA to increase the secretion of inflammatory cytokines and growth factors, leading to cartilage degeneration and destruction (25). This also suggests that the discovery of the MC subpopulation might provide new direction and value to the exploration of arthritic disease mechanisms (26). The IrC subpopulation was distributed in the whole cartilage layer, considered to be chondrocytes with inflammatory characteristics like immune cell adhesion, cytokine activity, and receptor. Leukocyte chemotactic genes were highly expressed in this subpopulation and these genes were enriched for the regulation of leukocyte cell-cell adhesion, immunological synapse, cytokine activity, and cytokine-cytokine receptor interaction. The KEGG results suggested a relationship between the NF-Kappa B signaling pathway and RA disease. It has been proposed that NF-kB was associated with bone erosion and the progression of RA disease, exhibiting high levels of inflammatory cytokines such as IL-1, TNF-α, and IL-6, and was believed to be one of the major inflammatory pathways in RA (27). Nuclear factor kappa B ligand (RANKL) was associated with the activation of NF-kB, which could lead to bone erosion and bone destruction, and the use of RANKL inhibitors could inhibit bone loss in RA by interfering with osteoclasts (28).

Based on the properties and functions of MC and IrC in the immune response, the analysis comparing the two cell subpopulations might improve our understanding of the role of chondrocytes in the pathogenesis of RA from an immunological perspective and even provide clues for RA cartilage regeneration. We performed a repopulation study of the two chondrocyte subpopulations, MC and IrC, and classified them into a total of three cell subpopulations, SELENOP+, IL32+, and SPP1+, based on the Marker gene of each subpopulation. Our study found that SELENOP+ cells and SPP1+ cells were mainly derived from MC, and Wang et al. also identified two macrophage clusters in immune cells, named SELENOP-Mφ and SPP1-Mφ (15), which also corroborated our definition of MC here. SELENOP protein is a transport carrier for the essential trace element selenium, which is mainly expressed in the liver and secreted into the plasma and has been shown to be associated with autoimmune diseases (29). SELENOP is also associated with oxidative stress, and the protein acts as an extracellular antioxidant with anti-inflammatory effects (30, 31). In addition, Wang et al. found that the SELENOP-Mφ cluster was also highly expressed in genes such as IL32, which participated in peptide metabolism, protein transport, and cytokine secretion, closely related to lymphocyte-related functions (15). SPP1, also known as OPN, has been shown to be involved in Mφ polarization and osteoclast attachment to the mineralized bone matrix, and also to promote chondrocyte proliferation through HOTAIR overexpression (32, 33). Knockdown of OPN could upregulate the expression of OA-related genes, and enhance chondrocyte senescence and apoptosis, accelerating the progression of OA (34). Increased expression of IL32-encoded cytokines in RA synovium induces pro-inflammatory cytokine expression, which was highly correlated with the severity of inflammation, suggesting it might be a potential therapeutic target for RA (35). In addition, unlike the induction of TNF-α, IL-1β and IL-6 via p38-MAPK, IL-32 was able to induce monocytes to differentiate into macrophage-like cells through a non-apoptotic, caspase-3-dependent mechanism, suggesting that IL-32 not only participated in the host response by inducing pro-inflammatory cytokines, but also directly affected specific immunity by differentiating monocytes into macrophage-like cells (36).

The presence of mechanical loading would affect the inflammatory state and growth factor expression of chondrocytes and interfere with chondrocyte proliferation and migration (37), but the effects of mechanical loading on chondrocyte types remain underresearched. This study revealed a significantly larger proportion of RegCs in the non-weight-bearing region, and that RegCs with high expression levels of chondroprotective genes might deserve further in-depth study. Notably, the MCs in the Fb group were mainly associated with antigen processing and presentation and MHC class II protein complex binding, which was consistent with more severe cartilage damage in the weight-bearing region of RA patients. The MCs in the Fnb group were mainly enriched for activities such as cartilage development and extracellular matrix organization, suggesting that cartilage in non-weight-bearing regions of RA patients might have more activated cartilage growth and development. Considering that the mechanical loading in OA could affect chondrocyte pyroptosis by influencing macrophage polarization (38), it is strongly reasonable to speculate that there is a process of macrophage polarization transition in MCs in response to mechanical loading, as in macrophages. MCs in the weight-bearing region are mainly involved in activities such as initiation and maintenance of immune response, and promotion of antigen processing and presentation, which are similar to the function of pro-inflammatory macrophages(M1) (39). In contrast, MCs in non-weight-bearing region are associated with activities such as connective tissue and cartilage development, which may have similar functions such as inflammation relieving and repairing like anti-inflammatory macrophages(M2) (39). Intervening in this regulation of immune homeostasis might be a good inspiration for decelerating the progression of RA, which could provide a reference for gene regulation or extraction of relatively high-quality cartilage from non-weight-bearing regions for targeted transplantation to reverse cartilage damage in weight-bearing regions of RA.

There are still some limitations to our study. First, single-cell sequencing is expensive, the number of samples taken in this study was limited, and there is still space for improvement in sampling technique and sample quality. Secondly, the samples were taken from both weight-bearing and non-weight-bearing areas of RA cartilage, and the lack of controls with healthy groups might result in less significant differences between the groups, and in-depth analysis and validation with multiple samples of healthy cartilage would make the results more reliable. Thirdly, we have not conducted in-depth *in vivo* and *in vitro* experiments to validate our findings and the practical clinical value of the results of this study needs to be further explored.

## 5 Conclusions

In conclusion, our study dissociated and characterized RA chondrocytes at single-cell resolution and identified two new immune activities related chondrocyte subpopulations, MC and IrC, revealing the different functions of MCs under mechanical loading and specific markers as well as the key transcription factors involved in each cell subpopulation, providing new possibilities for the development of diagnostic and therapeutic strategies for RA.

## Data availability statement

The datasets presented in this study can be found in online repositories. The names of the repository/repositories and accession number(s) can be found below: PRJNA999858 (SRA).

## Ethics statement

The studies involving humans were approved by the Institutional Ethics Review Committee of the Affiliated Hospital of Qingdao University. The studies were conducted in accordance with the local legislation and institutional requirements. The participants provided their written informed consent to participate in this study.

## Author contributions

MY, ZS, and TW contributed to the conception and design of the study. MY wrote the manuscript. HZ collected cartilage samples. MY and JW performed data analysis. TW, TY, and YZ supervised the manuscript. All authors contributed to the manuscript revision, read, and approved the submitted version.
