# Supplementary figures and images for: Single-cell RNA sequencing reveals distinct chondrocyte states in femoral cartilage under weight-bearing load in Rheumatoid arthritis
Source: Front Immunol. 2023 Aug 16;14:1247355. doi: 10.3389/fimmu.2023.1247355 (PMC10467429; doi:10.3389/fimmu.2023.1247355)

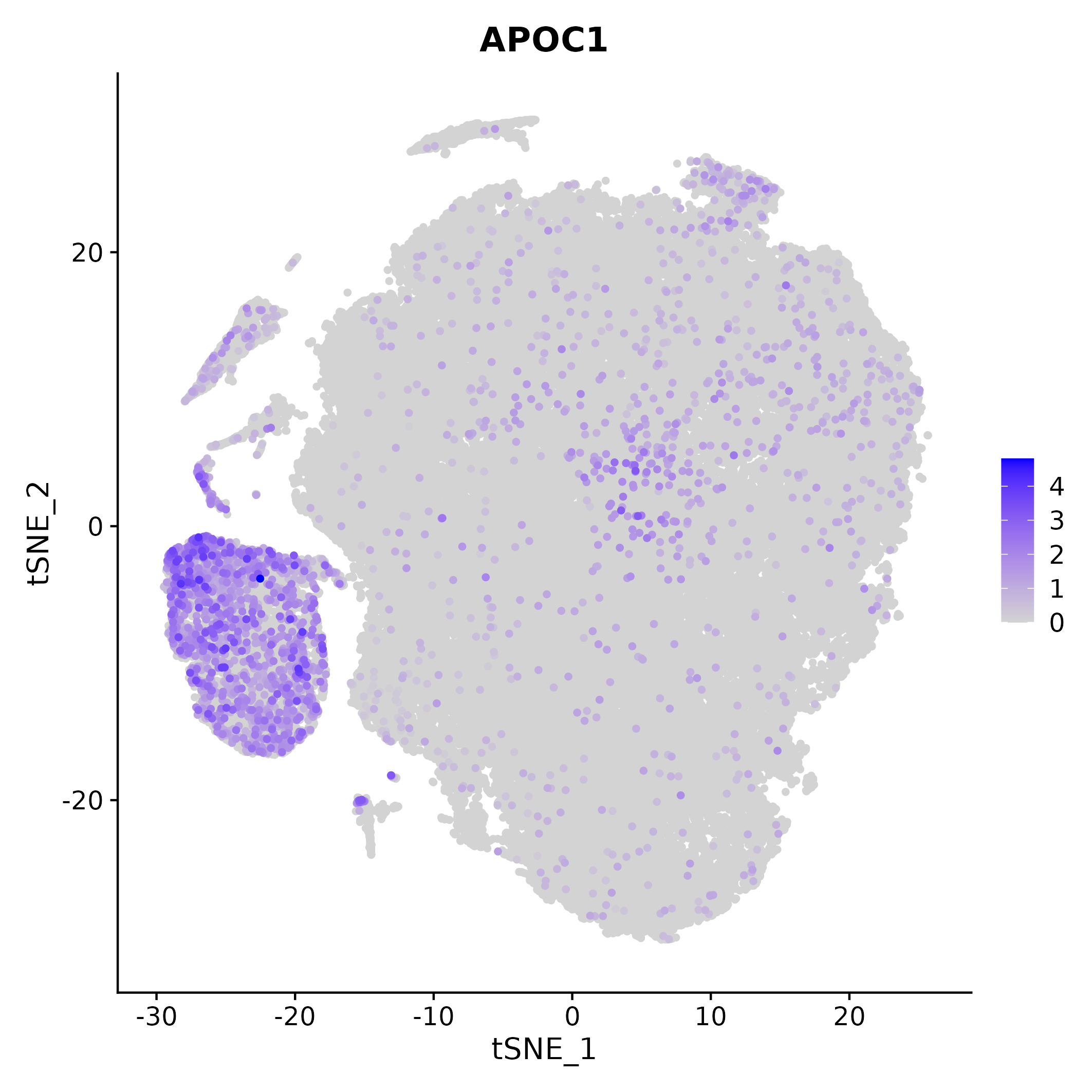

Supplement: Supplementary file 1 [file Image_1.tiff]

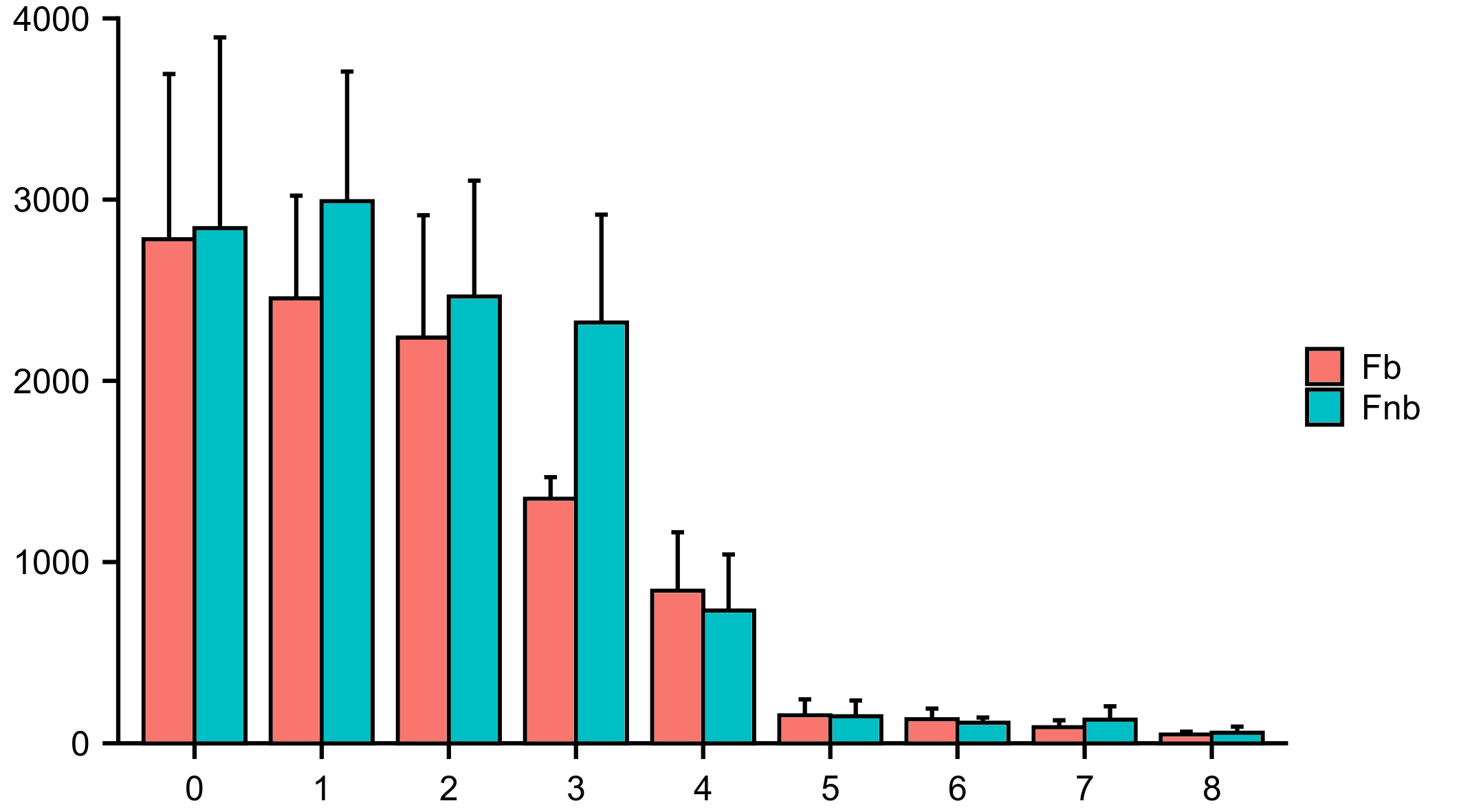

Supplement: Supplementary file 2 [file Image_2.tiff]

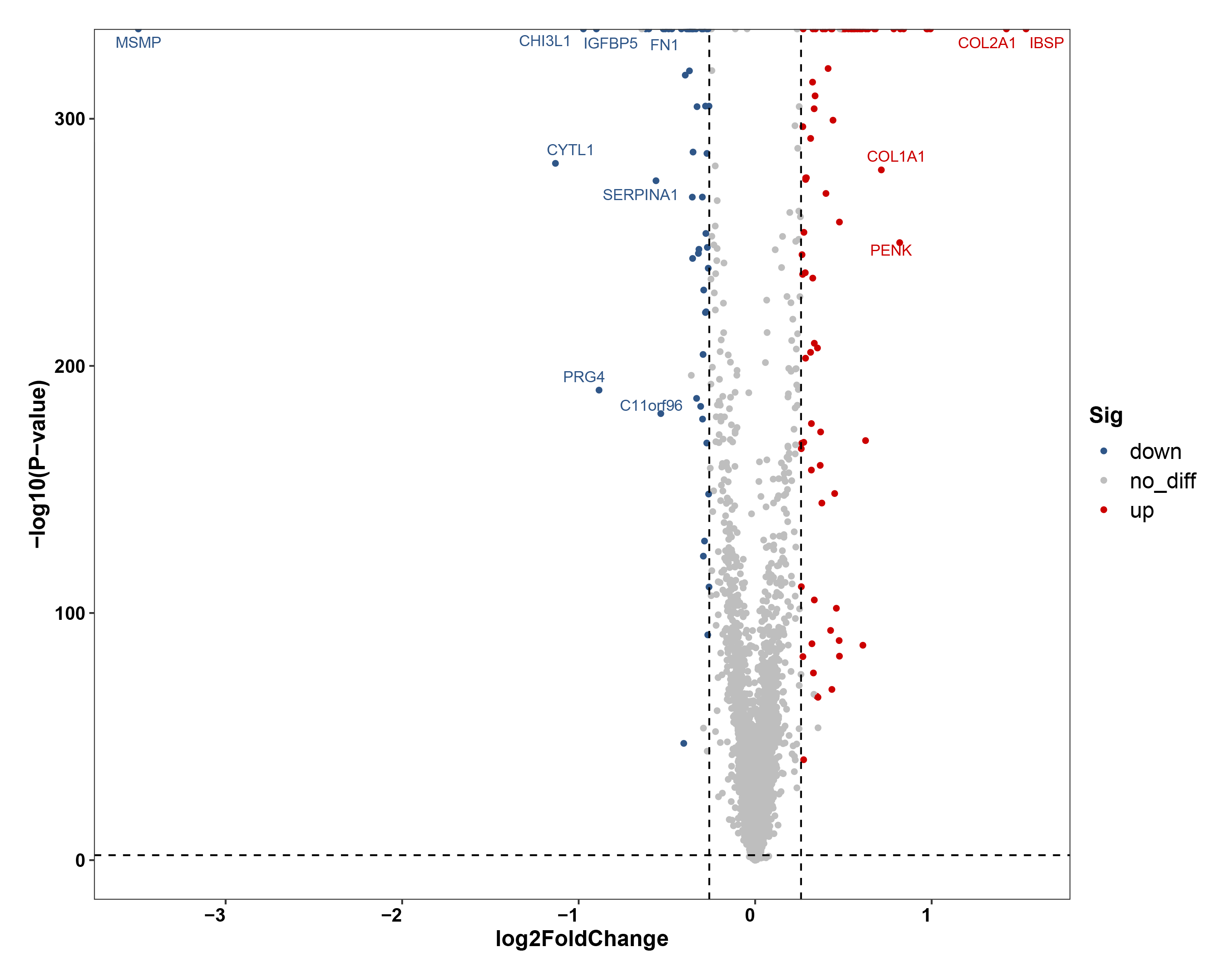

Supplement: Supplementary file 3 [file Image_3.tif]

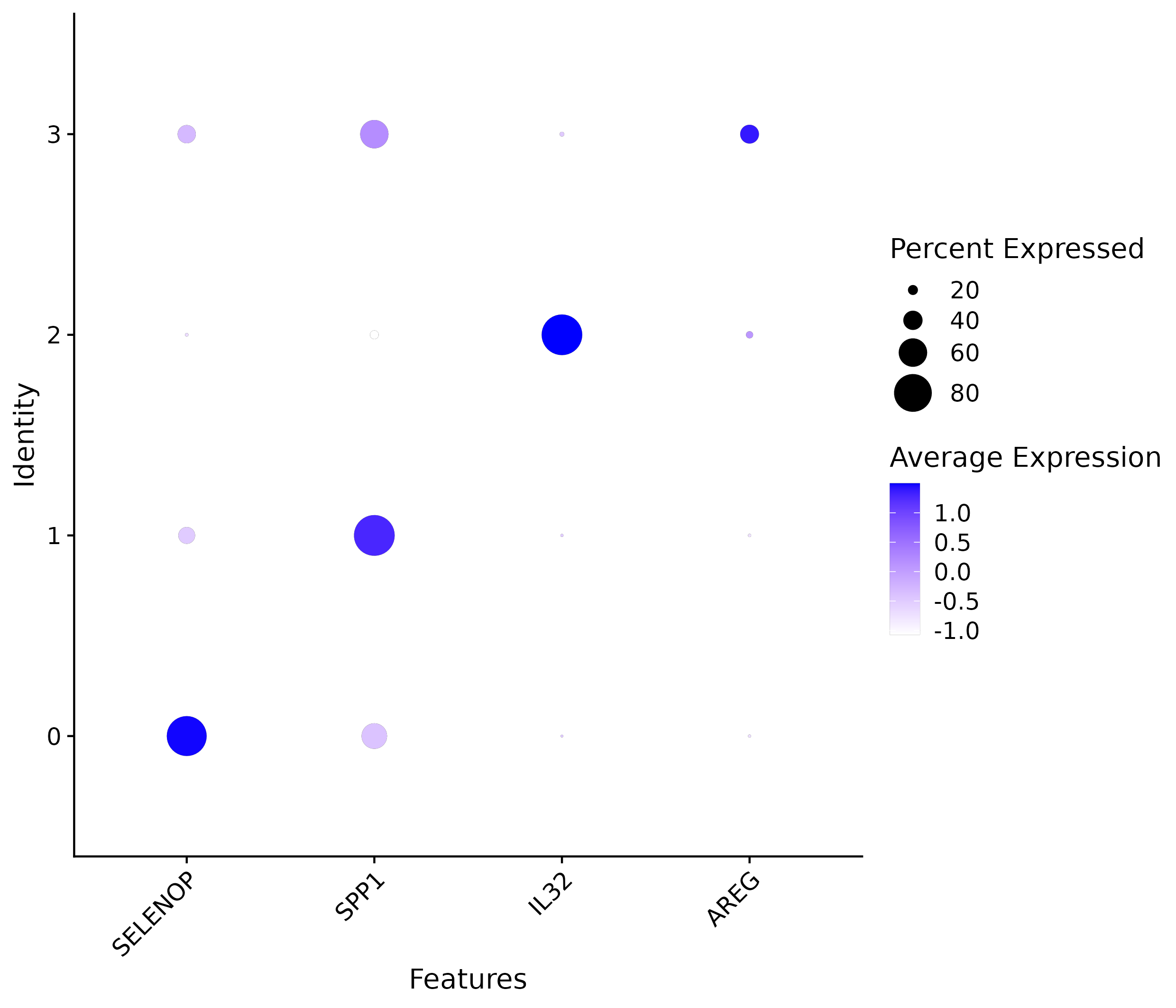

Supplement: Supplementary file 4 [file Image_4.tiff]

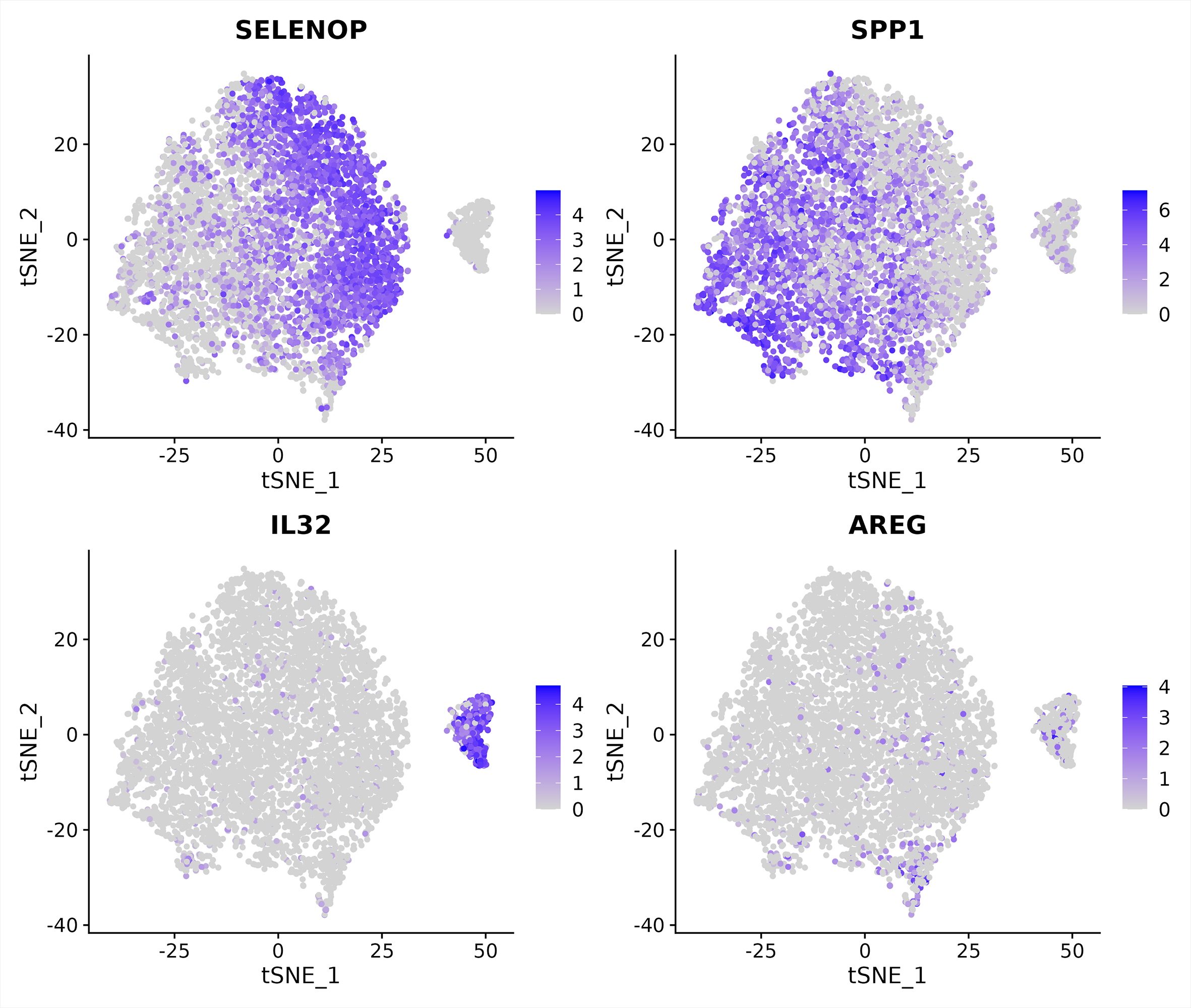

Supplement: Supplementary file 5 [file Image_5.tiff]
